# Supplementary material for: Intracranial Metastases from Uterine Leiomyosarcoma: A Systematic Review and Case Illustration
Source: J Clin Med. 2025 Sep 20;14(18):6631. doi: 10.3390/jcm14186631 (PMC12470581; doi:10.3390/jcm14186631)
Supplement: Supplementary file 1 [file jcm-14-06631-s001.zip › jcm-3817436-supplementary.pdf]

Table S1: Radiological features and diagnosis,

Author	Year	Age	Country	CT scan	MRI	Lung Mets	BM Presentation	BM Consistency	BM Location	BM No.	Time to BM (M)	EOR	Adjuv. for BM	Positive IHC Markers	Survival After BM (M)	Cause of Death
Vaquero et al.	1989	26	Spain	NS	NS	Yes	NS	NS	PAR	1	NS	T	Chemo	NS	50	CNS
Prussia et al.	1992	36	West Indies	Hyper	NS	NS	Coma	Fleshy/ Firm/ Hemorrhagic	PAR	1	72	T	No	NS	NS	NS
Wronski et al.	1994	60	USA	NS	NS	Yes	Seizure/ Monoparesis	NS	PAR	1	78	T	WBRT	NS	30	Systemic
Bindal et al.	1994	55	USA	NS	NS	Yes	NS	NS	CER	1	116	T	WBRT	NS	14	Systemic
Uchino et al.	1996	54	Japan	Osteolytic	HE	No	Bulging mass	Firm	FRO	1	48	T	Chemo	NS	24	Lung
Salvati et al.	1998	58	Italy	NS	NS	Yes	Low LOC	NS	FRO	1	3	T	WBRT	NS	3	NS
Salvati et al.	1998	43	Italy	NS	NS	Yes	Low LOC	NS	FRO	1	48	T	WBRT	NS	5	NS
Salvati et al.	1998	28	Italy	NS	NS	Yes	Low LOC	NS	FRO	1	26	T	WBRT	NS	5	NS
Ziyal et al.	1999	38	Turkey	Hyper/ Osteolytic	NS	Yes	Low LOC	Hemorrhagic	FRO	1	72	T	WBRT	NS	4	CNS
Mawrin et al.	2002	59	Germany	NS	Iso T1&T2, RE/CN/ Hemorrhagic	Yes	Diplopia/ Gait disturbances / Face hypesthesia	Hemorrhagic	PON	1	36	Bx	WBRT	Lam/ Vim	2	CNS

Yip et al.	2006	63	Taiwan	Osteolytic	Iso T1&T2/ HE	NS	Bulging mass/ Hemiplegia	Firm/ Hemorrhagic	PAR	1	15	T	No	Act/ Desm	4	Systemic
Munakata et al.	2006	52	Japan	NS	NS	No	Dysarthria/ Monoparesis	NS	CR	1	36	Bx	GKRS	NS	NS	NS
Melone et al.	2008	57	Italy	NS	RE/CN	Yes	Low LOC	Fleshy/ Firm/ Hemorrhagic	TEMP	1	12	T	WBRT+ Chemo	NS	Alive	___
Kaya et al.	2009	60	Turkey	NS	Hyper T2/ HE/CN	Yes	Diplopia/Facial & Abducens palsy	____	PON/ FRO	2	0	___	WBRT+ Chemo	Act/Vim/ Desm	NS	NS
Benizelos et al.	2009	51	Greece	NS	NS	Yes	Disequilibrium/ Nystagmus	____	CER	1	6	___	WBRT+ Chemo	Act/ Desm	3	Systemic
Pereira et al.	2011	55	Brazil	NS	Osteolytic/ HE/CN	No	Bulging mass	Firm	PAR	1	60	T	No	Act/ Desm	5	Systemic
Honeybul et al.	2009	42	Australia	NS	RE/CN/ DB	Yes	Blurry vision	NS	OCC	1	10	T	No	NS	2	Systemic
Venizelos et al.	2011	57	Greece	Hyper	RE/CN/ DB	Yes	Disequilibrium	Hemorrhagic	CER	1	8	STR	WBRT	Act/Vim/ Desm	1.5	Systemic
Yamada et al.	2011	50	Japan	Hyper	Iso T1&T2/ HE/CN/ DB	Yes	Headache	Hemorrhagic	OCC/ CER TEM/SPL	4	28	STR	GKRS	NS	12	Systemic
Mariniello et al.	2012	57	Italy	Hyper/ Osteolytic	HE/DB	No	Proptosis/ Hemiparesis	Firm/ Hemorrhagic	FRO/ ORB	1	8	STR	Chemo	Act/Vim	4	CNS
Chen et al.	2013	54	China	NS	NS	Yes	Seizure	____	PAR	1	48	___	GKRS+ Chemo	NS	At least 29	___
Shepard et al.	2014	54	USA	NS	NS	NS	Aphasia	____	FRO/PV	3	24	___	GKRS	NS	1	Systemic
Gurram et al.	2014	59	USA	Hyper	HE/DB	Yes	Lethargy/Slurred speech/ Hemiparesis	Fleshy/ Firm/ Hemorrhagic	CER/FRO	2	84	STR	SRS	NS	2	Systemic

Abrahao et al.	2015	45	Brazil	NS	NS	Yes	Hemiparesis	NS	PAR/ OCC/ FRO	3	31	T	WBRT+ Chemo	NS	27	NS
Abrahao et al.	2015	51	Brazil	NS	NS	Yes	Seizure	NS	PAR/C ER	3	22	T	WBRT+ Chemo	NS	16	Systemic
Kim et al.	2016	57	South Korea	Osteol ytic/ HE/CN	NS	Yes	Proptosis/Blu rry vision	Firm	TEP/F RO/ ORB	2	36	____	____	___	1	Systemic
Inoue et al.	2016	48	Japan	NS	Iso T1&2/ HE/CN/ DB	Yes	Hemiparesis/ Aphasia	NS	FRO	1	77	T	Chemo	Act/Vim Desm/ EMA	At least 18	___
Ahuja et al.	2017	60	India	Osteol ytic/ HE	Hyper T2/ HE/CN	Yes	Bulging mass	Firm	FRO	1	6	____	Chemo	Act/Vim/ pan-CK	Alive	___
Sosa et al.	2018	43	Argentin a	Hyper/ Osteol ytic	Iso T1/ Hyper T2/ HE/CN/ DB	Yes	Monoparesis	Firm	FRO	1	0	T	No	Act	1	Systemic
Chahdi et al.	2018	46	Morocco	NS	Hyper T2/ HE/DB	NS	Hemiparesis/ Hemiparesth esia	NS	FRO/P AR	1	84	T	WBRT	Act/Cald	NS	NS
Imoumby et al.	2021	46	Morocco	Hyper/ Osteol ytic	HE/DB	Yes	Hemiplegia	NS	PAR	2	60	T	WBRT	Act/ Vim/ Cald	5	Systemic
Miki et al.	2021	35	Japan	Hyper/ Osteol ytic	HE/DB	NS	Follow-up exam	NS	PAR	1	36	T	WBRT	NS	Alive	___
Miki et al.	2021	70	Japan	Hyper/ Osteol ytic	HE/DB	Yes	Follow-up exam	NS	TEM	1	96	T	No	NS	Alive	___
Miki et al.	2021	51	Japan	Hyper/ Osteol ytic	HE/DB	Yes	Follow-up exam	NS	PAR	1	48	T	WBRT	NS	Alive	___
Soo et al.	2022	60	Malaysia	Hyper/ Osteol ytic/H E/SDH	NS	No	Bulging mass	Firm	FRO	1	0	T	WBRT+ Chemo	NS	Alive	___

Delgado et al.	2022	33	Mexico	NS	Hyper T2/ HE/CN	Yes	Seizure	NS	OCC	1	10	T	WBRT+ Chemo	NS	NS	NS
Eatz et al.	2023	55	USA	NS	NS	Yes	Seizure	NS	FRO	1	5	Bx	SRS	NS	2	Systemic
Richards et al.	2023	51	USA	NS	Hyper T2/ HE/CN	Yes	Blurry vision	NS	TEM/ OCC	2	44	T	Chemo+ SRS	Myo	Alive	___
Carrilo-Uzeta et al.	2025	46	Mexico	Osteolytic	HE/CN/ DB	No	Hemiparesis	Firm/ Hemorrhagic	PAR	1	0	T	WBRT	SMA/ Cald	At least 12	___
Our Case	2025	49	USA	Hyper	Hypo T1/ Hyper T2/ HE/CN/ DB	Yes	Monoparesis	___	FRO/SPL	6	2	____	GKRS	Act/Vim	20	Systemic

*EOR: extent of resection; M: months; BM: brain metastasis; Mets: metastasis, Chemo: chemotherapy; SMA: smooth muscle antigen; EMA: epithelial membrane antigen; WBRT: whole brain radiotherapy; GKRS: gamma-knife radiosurgery; LOC: level of Consciousness; NS: not specified; Hyper: hyperdense/hyperintense; Iso: isointense; HE: Homogenous Enhancement; CN: Central Necrosis; RE: Ring Enhancement; SDH: subdural hematoma; Bx: biopsy; T: total removal; STR: subtotal tumor removal; DB: dural-based; Lam: laminin; Act: actin; Vim: vimentin; Desm: desmin; Cald: h-caldesmon; Myo: myosin; Alive: alive at the time of publication

Table S2: Search strings,

**PubMed**
**("Uterine Neoplasms"[Mesh] OR uterine OR uterus OR endometr* OR corpus uteri) AND ("Leiomyosarcoma"[Mesh] OR leiomyosarcoma* OR "LMS" OR "leiomyosarcom*") AND ("Brain Neoplasms"[Mesh] OR "Central Nervous System Neoplasms"[Mesh] OR brain OR intracran* OR intracerebr* OR intracranial OR cerebral OR cerebellar OR metastas* OR "brain metastasis" OR "central nervous system metastasis" OR leptomeningeal OR dural OR meningeal)**
("Uterine Neoplasms"[MeSH Terms] OR ("uterin"[All Fields] OR "uterines"[All Fields] OR "uterus"[MeSH Terms] OR "uterus"[All Fields] OR "uterine"[All Fields]) OR ("uterus"[MeSH Terms] OR "uterus"[All Fields] OR "uteri"[All Fields]) OR "endometr*"[All Fields] OR ("corpus"[All Fields] AND ("uterus"[MeSH Terms] OR "uterus"[All Fields] OR "uteri"[All Fields]))) AND ("Leiomyosarcoma"[MeSH Terms] OR "leiomyosarcoma*"[All Fields] OR "LMS"[All Fields] OR "leiomyosarcom*"[All Fields]) AND ("Brain Neoplasms"[MeSH Terms] OR "Central Nervous System Neoplasms"[MeSH Terms] OR ("brain"[MeSH Terms] OR "brain"[All Fields] OR "brains"[All Fields] OR "brain s"[All Fields]) OR "intracran*"[All Fields] OR "intracerebr*"[All Fields] OR ("intracranial"[All Fields] OR "intracranially"[All Fields]) OR ("cerebrally"[All Fields] OR "cerebrum"[MeSH Terms] OR "cerebrum"[All Fields] OR "cerebral"[All Fields] OR "brain"[MeSH Terms] OR "brain"[All Fields]) OR ("cerebellum"[MeSH Terms] OR "cerebellum"[All Fields] OR "cerebellar"[All Fields]) OR "metastas*"[All Fields] OR "brain metastasis"[All Fields] OR "central nervous system metastasis"[All Fields] OR ("leptomeninge"[All Fields] OR "leptomeningeal"[All Fields] OR "leptomeninges"[All Fields] OR "leptomeningitis"[All Fields]) OR "dural"[All Fields] OR ("meningeal"[All Fields] OR "meninges"[MeSH Terms] OR "meninges"[All Fields] OR "meninge"[All Fields] OR "meningism"[MeSH Terms] OR "meningism"[All Fields] OR "meningisms"[All Fields] OR "meningitis"[MeSH Terms] OR "meningitis"[All Fields] OR "meningitides"[All Fields]))
**Translations**
**uterine:** "uterin"[All Fields] OR "uterines"[All Fields] OR "uterus"[MeSH Terms] OR "uterus"[All Fields] OR "uterine"[All Fields]
**uterus:** "uterus"[MeSH Terms] OR "uterus"[All Fields] OR "uteri"[All Fields]
**uteri:** "uterus"[MeSH Terms] OR "uterus"[All Fields] OR "uteri"[All Fields]

**brain:** "brain"[MeSH Terms] OR "brain"[All Fields] OR "brains"[All Fields] OR "brain's"[All Fields]<br><br>**intracranial:** "intracranial"[All Fields] OR "intracranially"[All Fields]<br><br>**cerebral:** "cerebrally"[All Fields] OR "cerebrum"[MeSH Terms] OR "cerebrum"[All Fields] OR "cerebral"[All Fields] OR "brain"[MeSH Terms] OR "brain"[All Fields]<br><br>**cerebellar:** "cerebellum"[MeSH Terms] OR "cerebellum"[All Fields] OR "cerebellar"[All Fields]<br><br>**leptomeningeal:** "leptomeninge"[All Fields] OR "leptomeningeal"[All Fields] OR "leptomeninges"[All Fields] OR "leptomeningitis"[All Fields]<br><br>**meningeal:** "meningeal"[All Fields] OR "meninges"[MeSH Terms] OR "meninges"[All Fields] OR "meninge"[All Fields] OR "meningism"[MeSH Terms] OR "meningism"[All Fields] OR "meningisms"[All Fields] OR "meningitis"[MeSH Terms] OR "meningitis"[All Fields] OR "meningitides"[All Fields]
**Web of Science**
**("Uterine Neoplasms"[Mesh] OR uterine OR uterus OR endometr* OR corpus uteri) AND ("Leiomyosarcoma"[Mesh] OR leiomyosarcoma* OR "LMS" OR "leiomyosarcom*") AND ("Brain Neoplasms"[Mesh] OR "Central Nervous System Neoplasms"[Mesh] OR brain OR intracran* OR intracerebr* OR intracranial OR cerebral OR cerebellar OR metastas* OR "brain metastasis" OR "central nervous system metastasis" OR leptomeningeal OR dural OR meningeal)**
**Embase**
**('uterus'/exp OR uterine:ab,ti OR uterus:ab,ti OR 'corpus uteri':ab,ti) AND ('leiomyosarcoma'/exp OR leiomyosarcoma*:ab,ti OR lms:ab,ti) AND ('brain'/exp OR 'central nervous system'/exp OR brain:ab,ti OR intracran*:ab,ti OR intracerebr*:ab,ti OR intracranial:ab,ti OR cerebral:ab,ti OR cerebellar:ab,ti OR 'brain metastasis':ab,ti OR 'cns metastas*':ab,ti OR leptomeningeal:ab,ti OR dural:ab,ti OR meningeal:ab,ti)**
**Google Scholar**
**("uterine leiomyosarcoma" OR "uterine LMS" OR "leiomyosarcoma uterus") "brain metastasis" OR "intracranial metastasis" OR "CNS metastasis"**

Table S3: JBI Case Reports Appraisal,

<u>JBI Case Reports Appraisal</u>	Vaquero et al., 1989	Prussia et al., 1992	Wronski et al., 1994	Bindal et al., 1994	UchiN et al., 1996	Salvati et al., 1998	Ziyal et al., 1999	Mawrin et al., 2002	Yip et al., 2006	Chen et al., 2013	Shepard et al., 2014	Gurram et al., 2014	Munakata et al., 2006	Melone et al., 2008	Kaya et al., 2009	Benizelos et al., 2009	Pereira et al., 2011
Were patient’s demographic characteristics clearly described?	Y	Y	Y	Y	Y	Y	Y	Y	Y	Y	Y	Y	Y	Y	Y	Y	Y
Was the patient’s history clearly described and presented as a timeline?	N	Y	Y	Y	Y	Y	Y	Y	U	Y	N	Y	Y	Y	Y	Y	Y
Was the current clinical condition of the patient on presentation clearly described?	Y	N	Y	Y	Y	Y	Y	Y	Y	Y	Y	Y	Y	Y	Y	Y	Y
Were diagNstic tests or assessment methods	N	U	N	N	U	N	U	Y	Y	N	N	Y	N	Y	Y	N	Y

and the results clearly described?																	
Was the intervention(s) or treatment procedure(s) clearly described?	Y	Y	Y	Y	Y	Y	Y	Y	Y	Y	Y	Y	Y	Y	Y	Y	Y
Was the post-intervention clinical condition clearly described?	Y	N	Y	Y	Y	Y	Y	Y	Y	Y	Y	Y	N	Y	N	Y	Y
Were adverse events (harms) or unanticipated events identified and described?	Y	N	Y	Y	Y	Y	Y	Y	Y	Y	Y	Y	N	Y	N	Y	Y
Does the case report provide takeaway lessons?	Y	Y	Y	Y	Y	Y	Y	Y	Y	Y	Y	Y	Y	Y	Y	Y	Y
Total score (out of 8 points)<br>(Y=1, N=0, U=0.5, N/A=0)	6	4.5	7	7	7.5	7	7.5	8	7.5	7	6	8	5	8	6	7	8

JBI Case Reports Appraisal	Honeybul et al., 2009	Venizelos et al., 2011	Yamada et al., 2011	Mariniello et al., 2012	Sosa et al., 2018	Chahdi et al., 2018	Delgado et al., 2022	Eatz et al., 2023	Richards et al., 2023	Carrilo-Uzeta et al., 2025	Abrahao et al., 2015	Kim et al., 2016	INue et al., 2016	Ahuja et al., 2017	Miki et al., 2021	Soo et al., 2022	Imoumby et al., 2021
Were patient’s demographic characteristics clearly described?	Y	Y	Y	Y	Y	Y	Y	Y	Y	Y	Y	Y	Y	Y	Y	Y	Y
Was the patient’s history clearly described and presented<br><br>as a timeline?	Y	Y	Y	Y	Y	N	Y	Y	Y	Y	Y	Y	Y	Y	U	Y	Y
Was the current clinical condition of the patient on presentation clearly described?	Y	Y	Y	Y	Y	Y	Y	Y	Y	Y	Y	Y	Y	Y	Y	Y	Y
Were diagNstic tests or assessment methods	Y	Y	Y	Y	Y	Y	Y	N	Y	Y	N	U	Y	Y	Y	U	Y

and the results clearly described?																	
Was the intervention(s) or treatment procedure(s) clearly described?	Y	Y	Y	Y	Y	Y	Y	Y	Y	Y	Y	N	Y	Y	Y	Y	Y
Was the post-intervention clinical condition clearly described?	Y	Y	N	Y	Y	N	N	Y	Y	Y	Y	N	Y	Y	Y	Y	Y
Were adverse events (harms) or unanticipated events identified and described?	Y	Y	Y	Y	Y	N	N	Y	Y	Y	Y	Y	Y	Y	Y	Y	Y
Does the case report provide takeaway lessons?	Y	Y	Y	Y	Y	Y	Y	Y	Y	Y	Y	Y	Y	Y	Y	Y	Y
Total score (out of 8 points)<br><br>(Y=1, N=0, U=0.5, N/A=0)	8	8	8	8	8	5	6	7	8	8	7	5.5	8	8	7.5	7.5	8
